# Gazing the dusty mirror: Joint effect of narcissism and sadism on workplace incivility *via* indirect effect of paranoia, antagonism, and emotional intelligence

**DOI:** 10.3389/fpsyg.2022.944174

**Published:** 2022-08-02

**Authors:** Bo Wang, Muhammad Fiaz, Yasir Hayat Mughal, Alina Kiran, Irfan Ullah, Worakamol Wisetsri

**Affiliations:** ^1^School of Management and Economics, Beijing Institute of Technology, Beijing, China; ^2^Research Centre for Sustainable Development & Intelligent Decision, Beijing Institute of Technology, Beijing, China; ^3^Department of Management Science, Qurtuba University of Science and Information Technology, Dera Ismail Khan, Pakistan; ^4^Department of Health Administration, College of Public Health and Health Informatics, Qassim University, Buraydah, Saudi Arabia; ^5^Department of Technology and Management, Universiti Teknikal Malaysia Melaka, Malacca, Malaysia; ^6^School of Management and Economics, Beijing Institute of Technology, Beijing, China; ^7^Department of Social Science, Faculty of Applied Arts, King Mongkut's University of Technology North Bangkok (KMUTNB), Bangkok, Thailand

**Keywords:** narcissism, workplace incivility, paranoia, sadism, antagonism, emotional intelligence

## Abstract

Workplace productivity is badly affected by many negative factors such as narcissism, and sadism. In addition, paranoia and antagonism play an important role in increasing workplace incivility. Through emotional intelligence, such negative behaviors could be addressed by managers and their junior colleagues. The current study aims to investigate the parallel mediating role of paranoia, antagonism, and emotional intelligence on the relationship between narcissism, sadism, and workplace incivility. A survey approach was used. Primary data was collected in PLS-SEM. The population of the study was all faculty members in higher education institutions in the Khyber Pakhtunkhwa (Pakistan) region. A measurement model and structural model were developed. The measurement model demonstrated that convergent and discriminant validities were established. The structural model's findings revealed that narcissism, antagonism, and emotional intelligence were not mediated between narcissism and workplace incivility. Similarly, emotional intelligence did not play any mediating role between sadism and workplace incivility. This implied that emotional intelligence has no role in decreasing or reducing workplace uncivil behavior.

## Introduction

Facing uncivil behavior from executives and subordinates is common in organizations (Lim and Cortina, 2005). Uncivil behavior includes violation of workplace norms, ignoring subordinates or managers, demeaning their rights, and patronizing their self-esteem (Cortina et al., 2001). As a result of these activities, the workplace has become discourteous, which is defined as “low-intensity aberrant conduct with uncertain intent to harm the target.” Uncivil activities are generally unpleasant and disrespectful (Dion, 2006). Researchers are concerned about workplace incivility since it can lead to mental health issues and a loss of enthusiasm for one's job (Bunk and Magley, 2013). Previous studies found that both job and personality variables influence the development of counterproductive work behavior (CWB) (Hershcovis et al., 2007). CWB comprises disrespecting employees and subordinates or focusing on oneself (e.g., Spector et al., 2006). As workplace incivility is a sub-form of CWB (Cortina et al., 2001), previous research has found that both work characteristics and personality factors have a substantial effect on the development of CWB (Hershcovis et al., 2007). Further, some researchers have discovered a link between narcissism and CWB. Narcissism may have a role in subtle workplace behavior like incivility (Judge et al., 2006; Edwards and Greenberg, 2010). Narcissists need to be addressed differently since they have a skewed self-image and grandiose emotions. That is why they constantly seek acceptance to validate their inflated self-view (Twenge and Campbell, 2003). Incivility in the workplace is more common among narcissists who feel they don't get their due. Similarly, sadism is defined as deliberately seeking opportunities to practice and enjoy cruelty (Plouffe et al., 2019), and it can range from moderate attitudes to pathological behavioral manifestations (O'Meara et al., 2011). As a result, sadists like breaking organizational standards and engaging in interpersonal deviance. After adjusting for the dark triad, sadistic employees are more likely than non-sadistic employees to engage in interpersonal deviance that ultimately induces mental health issues (Min et al., 2019).

However, psychological trauma induced by unpleasant prior social encounters, long-term harassment, and childhood maltreatment leads to paranoia cognition (Freeman et al., 2002). Unpleasant sentiments might lead to paranoid thinking processes (Chan and McAllister, 2014). Indeed, self-consciousness is associated with paranoid arousal, which is characterized by dread, worry, and a sense of possible danger, as well as low mood, especially discomfort (Gracie et al., 2007). It is important to note that antagonism comprises a wide range of negative personality traits that lead to conflict with others. As a tendency or in its specific forms, antagonism is dependent on interpersonal or societal dynamics (Pincus and Ansell, 2013; Fatfouta et al., 2017; Iñiguez and Lietor, 2021). Maladaptive or aggressive personalities frequently engage in interpersonal conflict in social situations. The basic dimensions of antagonistic persons include manipulativeness, callousness, disagreeableness, and deceitfulness (Vize et al., 2019, 2020).

Furthermore, to address the gap in an existing body of knowledge and literature on workplace incivility, by bridging two theories—contemporary integrative interpersonal theory (Sullivan, 2013), and the polyvagal theory (Porges, 1995)—we have developed a complex mediation model that explains why individuals with dark personality traits i.e., narcissism and sadism as a core of the dark tetrad (Hilbig et al., 2021) with some stigmatized identities (paranoia and antagonism) lead to emotional impairment. How do common triggers of individuals' paranoia play out in an organizational setting? Can paranoia be reasonable? Similarly, how to deal with the core characters of dark tetrad i.e., sadism or narcissism, if they possessed antagonistic personality evils? How to identify their hidden prevalence? Through emotional intelligence, can we deal with these hidden identities? None of the previous studies have examined paranoid personality disorder and antagonistic manifestation in the context of the subject matter stressed here. However, the issue of whether short- or long-term antagonism can adequately account for the relationships they have with one another, and the impact of aggressive behavior on key outcomes remains unanswered. This has allowed us to investigate the commonness of paranoia and antagonistic behavior in the setting of narcissism and sadistic personality disorder, as paranoid personality disorder and antagonistic attitude have long-lasting implications in the professional workspace (Chan and McAllister, 2014; Fatfouta et al., 2017). Therefore, the current study examined the parallel mediation effect of paranoia and antagonism manifestation of narcissists and sadistic workers toward workplace incivility along with the lack of emotional intelligence. It aids us in the long-term process of addressing the root causes of workplace incivility issues. After more than a decade of research, researchers (Hurtz and Donovan, 2000; González-Morales et al., 2006; Min et al., 2019; Shiverdecker and LeBreton, 2019; Lopes et al., 2020), still haven't come up with any solutions that are worth using. We must first address an issue with the operationalization of the dark tetrad features at workplace abnormal outcomes before moving forward. They may be linked to personality disorders such as antagonism and paranoia, which are grounded in non-clinical psychology. Organizational psychologists have been studying antagonism, paranoia, and emotional intelligence (EI) under numerous identities for many years now. Incivility in the workplace is widely acknowledged to be a serious issue that hurts all those involved. Consequently, it is necessary to ascertain a solution to cease such negative behavioral patterns. Studying workplace incivility offenders will benefit firms. Workplace incivility should be examined to identify personality traits, which in turn can inform the recruiting process and develop a healthy work environment. Mental health practitioners can influence abusers' behavior in several ways. Throughout this article, we aim to provide a bird's eye view of the workplace hedonic forest.

Consequently, the current study makes a novel contribution to the literature and the theoretical constructs as follows:

Studies on narcissism, sadism and workplace incivility are limited.

Studies on antagonism, paranoia and emotional intelligence are a novel addition.

In the Pakistani context, empirical evidence is a novel contribution that has to be further developed.

## Theoretical overview and hypothesis development

### Theoretical basis

The current study was conducted on the bases of two personality theories. The contemporary integrative interpersonal theory (Sullivan, 2013), and the polyvagal theory (Porges, 1995). The CIIT is a scientifically based personality model that links an empirically determined structure to dynamic interpersonal, emotional, and behavioral processes to provide testable hypotheses regarding individual differences and situational behavior. The interpersonal circumplex is a two-dimensional model used by CIIT to define and measure interpersonal functioning (Fournier and Avery, 2011, p. 58). It also serves as a framework for integrating theories of personality, motivation, cognition, behavior, and psychopathology (Hopwood et al., 2013). A key feature of the CIIT concept of interpersonal interaction is that it encompasses both direct and indirect mental representations of ourselves and others (Tudor-Locke et al., 2011). How one perceives other people and how one expects others to respond to one's actions are significant aspects of interpersonal relationships (Pincus and Wright, 2011). Interpersonal circumstances represent an individual's interaction techniques, regulation functioning, and self-concept.

Similarly, the polyvagal theory described the evolutionary model of the autonomic nervous system (see Porges, 2003), and provides a unique theoretical framework for examining the parasympathetic nervous system's probable role in borderline personality disorder (BPD). The idea stresses the role of the autonomic state in influencing prosocial and defensive behavior—an integrated social engagement system (e.g., gaze, emotion, prosody, and gesture) (Winhall, 2021). This theory explains how autonomic state control brain circuits evolved to permit adaptive biobehavioral responses to stresses (Chase, 2021). Thus, issues in emotional regulation associated with BPD may be seen as a behavioral representation of a physiological condition that has evolved to support protective techniques in dangerous and life-threatening situations (Porges, 2003). A brain process that permits individuals to engage in social activities by distinguishing safe from dangerous circumstances is called “neuroception” in polyvagal theory (Porges, 2009). Hence, from the theoretical perspective, we integrate research on dark emotions (narcissism and sadism), and workplace incivility, with a mediation model of antagonism, and paranoid cognition that explains why employees at the workplace lead to emotional exhaustion and workplace incivility.

### Workplace incivility

Incivility is more likely to occur at work due to minimal interpersonal deviation (Lim and Cortina, 2005). Incivility at work increases negative feelings, job dissatisfaction, mental/physical health, absenteeism, and turnover intentions (Porath and Pearson, 2012). Low-level incivility has the potential to escalate into deliberate revenge behaviors (Lim et al., 2008). With a terrible personality, it's simpler to get away with bad behavior. The dark triad predicts incivility if sadists actively enjoy others' misery (Roberts et al., 2011). In the workplace, incivility is a regular occurrence (Reio and Sanders-Reio, 2011). Incivility, on the other hand, has garnered far more attention in the last two decades than other more serious workplace interpersonal mistreatments like bullying and physical assault (Cortina, 2008). In “Tit for Tat” Andersson and Pearson (1999) proposed that incivility is a low-intensity interpersonal abuse (Porath and Pearson, 2012). Incivility has a range of detrimental effects on job satisfaction and health, as well as worry, sadness, and wrath, which contribute to absenteeism and disengagement (Miner et al., 2012). It impacts work performance, withdrawal behavior (Sliter et al., 2012), as well as citizenship behavior (Taylor and Kluemper, 2012), and CWB is on the rise (Sakurai and Jex, 2012). According to Gui et al. (2022), incivility in the workplace can drain employees' emotional resources, resulting in emotional exhaustion; but meaningful work is a critical cognitive resource that can offset this relationship.

### Narcissism and workplace incivility

Narcissism is a sub facet of dark tetrad connected to a wide range of psychological and emotional issues, including strain, dysfunctional problems, impaired working relationships (Miller et al., 2007; Crowe et al., 2019), risk-taking, self-centeredness, and aggressive self-view (Kealy et al., 2017). Many studies focus on personal narcissism since it displays people's desire to engage in a range of activities to preserve excessively favorable self-perceptions by unmasking real views (Morf and Rhodewalt, 2001; Harms and Spain, 2015). Narcissists see uncivil behavior as a danger to their goal of a positive self-image and are forced to defend themselves (Pincus et al., 2009). They may be more outraged than apologetic since they may blame incivility on factors other than their personality flaws. Because they are self-centered, narcissists rarely consider others challenging (Campbell, 2005). Moreover, Brunell et al. (2011) found that narcissism can impair a sense of guilt and they are extremely sensitive to criticism and respond violently to insults and bad comments (Brunell et al., 2011). Anger, impulsiveness, low empathy, and an exaggerated self-view are all hallmarks of narcissists that have been linked to unproductive workplace behavior (Holtzman et al., 2010; Meier and Semmer, 2012). Morf and Rhodewalt (2001) found a modest link between narcissism and workplace incivility. Fury and guilt were revealed to be positive mediators in the study by Liu et al. (2020) on narcissism and workplace incivility. Through the mediation of respect for norms, Moon and Morais (2022) found that covert narcissists are more prone to endure workplace incivility. Employees' experience of incivility at work is influenced by their self-esteem and their perceptions of respect for workplace norms. Hence, we proposed that:

Hypothesis 1 (H1): Narcissism has a positive effect on workplace incivility.

### Sadism and workplace incivility

A subclinical type of sadism called “everyday sadism” was recently confirmed by psychologists as the core of dark personality traits (Buckels et al., 2013). Those with a higher level of everyday sadism may seize opportunities to either see or inflict misery on others (Buckels, 2012; Buckels et al., 2013). Moreover, according to Thibault and Kelloway (2016), sadism had a moderate influence on the dark triad and counterproductive workplace behavior, and the dark triad lost predictive value over CWB when sadism was low. These findings suggest that sadism may play a part in the development of other negative tendencies in the workplace. Also, Min et al. (2019) revealed that sadism is an active pleasure of others' agony, may predict interpersonal deviance, inspire incivility, and escalate the prevalence of cyberbullying over the other dark triad. Based on the above findings, it appears to be particularly useful in predicting workplace incivility among sadistic personalities. Mushtaq and Rohail (2021) investigated the relationship between the dark tetrad and workplace bullying. Psychopathic and Machiavellian personalities appear to have a beneficial impact on workplace bullying behavior, however, narcissism and daily sadism were found to have insignificant associations. Thus, we proposed that:

Hypothesis 2 (H2): Sadism has a positive effect on workplace incivility.

### Paranoia and workplace incivility

Studies on paranoid behavior in organizations include notions about being attacked, wounded, persecuted, mistreated, and disparaged by wicked people within the corporation (Kramer, 2001, p. 6). It is a condition of active psychological tension and fear (i.e., heightened paranoia) that is defined as “uncomfortably uncomfortable” (Gracie et al., 2007). These are long-lasting feelings that require a lot of work to overcome. Concern for one's survival is linked to these feelings (Freeman et al., 2014). According to social psychology, paranoiacs feel they are being hurt and persecuted because the perpetrator aims to hurt them (Gracie et al., 2007; Van Quaquebeke, 2016). As stated in the behavioral model of psychopathology, paranoid characteristics are made up of various paranoid schemata (Lopes et al., 2020). Hence, paranoids distort social data processing. All these things contribute to psychopathology. Problems in social information processing contribute to depressive cognitions (Chan and McAllister, 2014). Psychological mistakes that (mis)attribute harmful intent to others' employment behaviors may generate paranoia in workers (Chan and McAllister, 2014).

Likewise, Lopes et al. (2020) link paranoia to workplace bullying perceptions and intents. The same study found a link between supervisory paranoia and workplace malfeasance that was only moderated by past stressors and negative psychological experiences. According to Mitelman et al. (2020), this may be especially true for those with paranoia in stigmatized jobs. They also show that awareness is required for the harmful process connecting social stress, paranoia, and poor occupational wellbeing. For example, paranoid thinking habits deplete mental resources (Chan and McAllister, 2014), contributing to workplace incivility and emotional tiredness (Guchait et al., 2019). Moreover, Fan et al. (2022) studied paranoid ideation and social function, study results proposed that symptoms of paranoia vary in severity and length, and both have an impact on social functioning. Improved interpersonal functioning is associated with less severe paranoia and a greater range of paranoid symptoms. To summarize, we proposed that those who experienced more prejudice in past have paranoid cognition and, as a result, emotional fatigue at work and reengage with the cause of their discomfort. Accordingly, we suggest the following study hypothesis:

Hypothesis 3 (H3): Paranoia has a positive effect on workplace incivility.

### Antagonism and workplace incivility

Vicious confrontations and aberrant and antisocial behavior are prevalent kinds of maltreatment (Vickers, 2006). An intentional disengagement from prescribed activities and employment commitments is common. Unhappiness and lack of inspiration may lead to job loss (Porath and Pearson, 2013). Negative qualities, such as low agreeability and high antagonistic tendencies, are often viewed as untrustworthy, dishonest, greedy, uncooperative, uncivil, and haughtiness in people (Cortina et al., 2013; Shiverdecker and LeBreton, 2019). Further, multiple meta-analytic investigations have indicated that antagonism and task performance have a moderate association.

On the other hand, the findings of Judge et al. (2015) showed that a more comprehensive analysis of antagonism, rather than a single broad component, could be better. They also emphasized the necessity for organizational researchers to investigate how this link can evolve as the nature of work changes. A slight but substantial negative correlation between trait antagonism and task performance was discovered in their study. These results are comparable to those of Hurtz and Donovan (2000), who found a negative relationship between antagonism and work engagement. These two meta-analytic studies reveal a slight negative relationship between trait antagonism and work engagement. For example, if activities grow increasingly interdependent and collaborative, how would trait antagonism affect task performance? Hence, we suggest:

Hypothesis 4 (H4): Antagonism has a positive effect on workplace incivility.

### Emotional intelligence (EI) and workplace incivility

Emotionally intelligent people can control their desires, postpone fulfillment, manage their emotions, and prevent their misery from influencing their thinking (Lim and Cortina, 2005; Nagler et al., 2014). Researchers have employed emotional intelligence to reduce negative feelings, job stress, and weariness (Görgens-Ekermans and Brand, 2012). It is possible that EI can help people deal with counterproductive workplace behavior and it's harder to elicit deviant or uncivil behavior among emotionally mature employees (Petrides et al., 2004; Ricciotti, 2016). In the study by Mayer et al. (2000) significant association between emotional intelligence and worker misbehavior was found (Kariuki et al., 2018). According to Khalid et al. (2016), emotionally intelligent people can better control their emotions and prevent harmful actions. While Jung and Yoon (2012) argued that employees without heightened EI are more likely to engage in CWBs.

Thus, EI, like any other resource that may regulate interpersonal and emotional skills, is worth considering (Cherry et al., 2012; Leiter et al., 2015). Interpersonal abuse appears to be linked to the inability to form positive workplace relationships (Kim and Qu, 2019) and general stress (González-Morales et al., 2006; Peiró, 2008). Ramsey-Haynes (2021) too investigated the association between EI and workplace incivility. All these attributes correlated negatively with workplace incivility. Organizational culture benefits from high EI and low incivility, yet employees lack self-awareness about their behaviors. Improving EI could help nurses engage more positively with patients and coworkers. Previous research shows that EI skill training programs improve EI. Thus, we propose the following research hypothesis:

Hypothesis 5 (H5): Emotional Intelligence has a negative effect on workplace incivility.

### Mediating role of paranoid personality disorder

The dark triad strives to dominate society by taking advantage of others (Paulhus, 2014; Thomaes et al., 2017). There are several examples of groups that seek to control society by exploiting individuals, such as narcissists and sadistic individuals. Subclinical paranoia (Frazier et al., 2017) is a prevalent feature in the general population. In micro-organizational research, the paranoid personality trait is underrepresented (Chan and McAllister, 2014). In many cases, firms avoid collaborating with employees who have the same negative personality trait (Spain et al., 2014; Wood and Dennard, 2017). The psychological mechanism of paranoia inhibiting proactive behavior is currently being debated (Frazier et al., 2017; Guchait et al., 2019; Bani-Melhem et al., 2020). Paranoia is a serious mental disorder in one's personality that might lead to clinical or non-clinical problems (Spain et al., 2016).

Decades of paranoia have led to long-term resentment and violent responses to praise (Edens et al., 2009; Freeman et al., 2012). It is connected to antisocial and paranoid personality disorders, while psychotic and narcissistic personalities have been connected to disengaged, as well as borderline personality disorders. Despite this, Lenzenweger (2018) define malignant narcissism as narcissism with paranoia, psychopathic tendencies, aggression, and sadism. Malignant narcissists demonstrate paranoia, rage, and harshness toward others. A statistical analysis by Sofra (2020) revealed two narcissistic personality disorder (NPD) phases. Paranoia seems to help malignant narcissists. Their skepticism and alertness help them identify hidden threats. Brutality and disregard for human rights are part of the complex layer of protection of neurotic sadism, and sadism, not despair, fuels narcissistic thought. Some of the studies have explored managing sad personnel. Until recently, management studies neglected paranoia (e.g., Chan and McAllister, 2014). As a result, we anticipate the following:

Hypothesis 6a (H6a): Narcissism and workplace incivility is positively mediated by paranoia.
Hypothesis 6b (H6b): Sadism and workplace incivility is positively mediated by paranoia.

### Mediating role of antagonism

Contrary to popular belief, few studies have examined the dark triad of personality traits to discover the direct relationships between workplace incivility and antagonistic personality traits (Paulhus and Williams, 2002; Maharana, 2019; Wissing and Reinhard, 2019). The lack of significance for narcissism may be due to subtle conceptual differences or behavioral variations in antagonism (Shiverdecker and LeBreton, 2019). Antagonism is the core claim for antisocial behavior and personality disorders including sadism and narcissism (Miller et al., 2017). Foulkes (2019) claims that antagonism poses as a narcissistic trait, but with a distinguished style of charm like sadism. Antagonism has two narcissistic levels: grandiose and vulnerable (Miller et al., 2017). According to Foulkes (2019), sadism with hedonistic enjoyment should be explored with antisocial, low self-control, and impolite personality qualities, Tiedens (2001) contends that workplace incivility can harm both employees and employers. Fear and grief rise in low-status individuals because the severity of the penalties varies based on the individual's status and circumstances. Examples of untrustworthy zero-sum thinking include competing narcissistic interests that can explain the antagonistic personality of narcissists (Rózycka-Tran et al., 2015). According to Lynam and Miller (2019) antagonism is the second most important factor linking neuroticism and satisfaction. Antagonism can also be connected to accident history, and victimization is linked to antagonism. Beckert and Ziegele (2020) conducted a study on the joint effect of personality traits and situational factors on the civility of news website viewers and revealed that sadistic personality traits drive incivility in attitude while deliberative attitude results in a high level of agreeableness and least extraversion. Therefore, we suggest the following study hypotheses:

Hypothesis7a(H7a): Narcissism and workplace incivility is positively mediated by antagonism.
Hypothesis 7b (H7b): Sadism and workplace incivility is positively mediated by antagonism.

### Mediating role of emotional intelligence

Some EI traits are “dark” or “maladaptive”, and emotional manipulation is the deliberate exploitation of emotional capacity (Austin and Colman, 2008; Ali et al., 2009; Petrides et al., 2011). However, narcissism has been linked to every aspect of social-emotional control. Narcissists lack affective empathy and struggle to understand others' feelings (Rauthmann and Kolar, 2013). So social and emotional abilities may be faked. Parallelism of the dark triad with other traits forms a tetrad (e.g., violent behavior, terrible honesty). Moreover, sadism can be applied to partners or strangers, with or without consent. Sadistic feelings and deeds include dominance, humiliation, enslavement, biting, burning, flogging, penetration with foreign objects, strangling, and physical mutilation (Warren and Hazelwood, 2002). Empathy can assist in forecasting workplace incivility and help to create a respectful and civil workplace for all employees regardless of age, ethnicity, or gender (Rastogi and Shukla, 2021). In addition to the above-mentioned studies, Mededović and Petrović (2015) and Paulhus and Dutton (2016) claim that dark personalities indicate low interest in workplace results (Lata and Chaudhary, 2020). Also, O'Boyle et al. (2015) discovered that dark personalities are more prone to CWBs such as violence, workplace incivility, victimization, and bullying (Wu and Lebreton, 2011). Workplace incivility, for example, moderated the link between emotional intelligence, unproductive workplace conduct, and turnover intentions (Schilpzand et al., 2016). Hence from the above, we proposed the following hypotheses:

Hypothesis 8a (H8a): Narcissism and workplace incivility is negatively mediated by EI.
Hypothesis 8b (H8b): Sadism and workplace incivility is negatively mediated by EI.

## Materials and methods

### Participants and procedure

**Figure 1:**
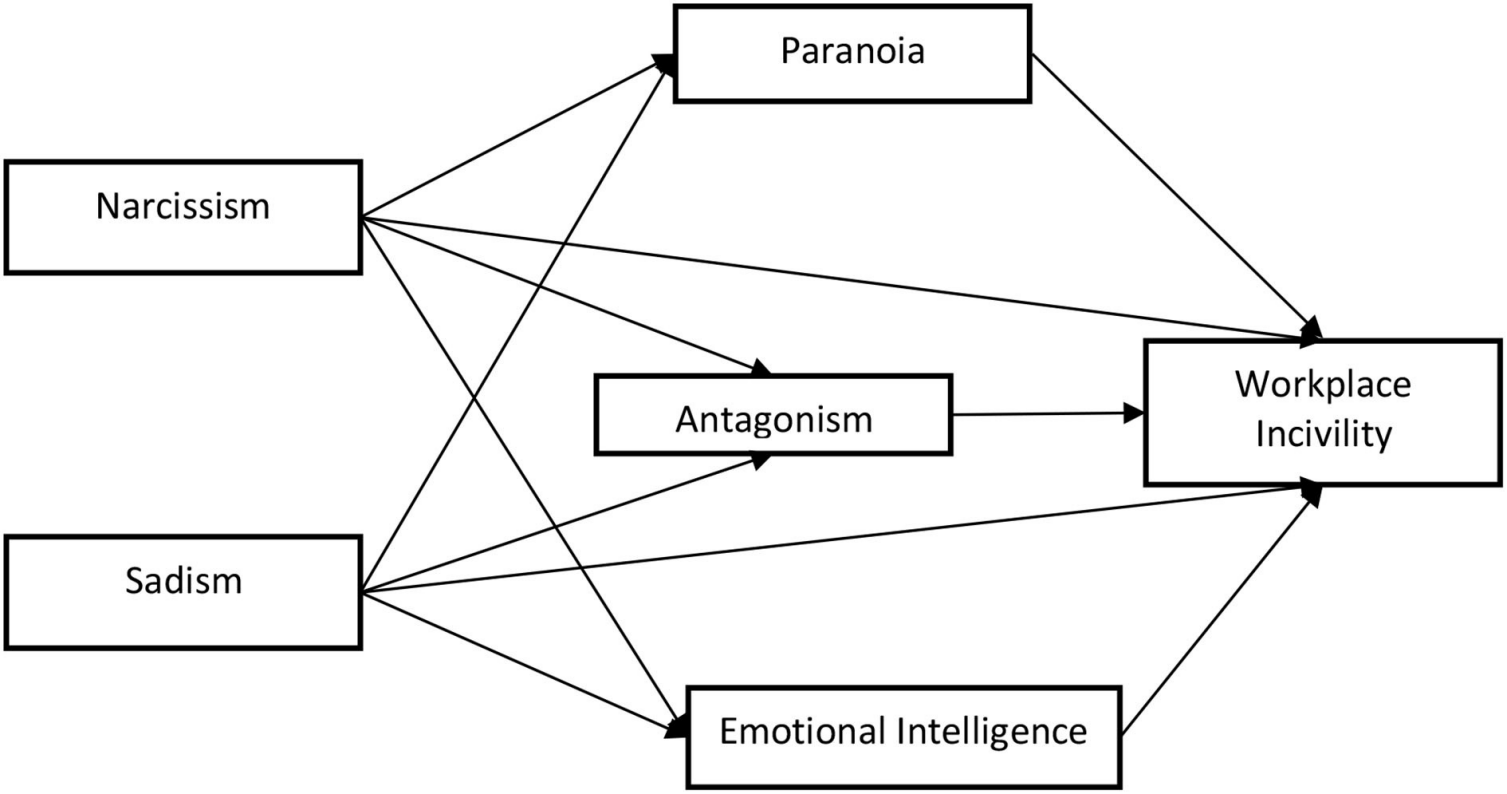
Theoretical framework.

The current study examined the relationship between narcissism, sadism, and workplace incivility, as well as the role of antagonism, paranoia, and emotional intelligence as mediating variables. The research model (Figure 1) was developed after conducting a study of the relevant literature. The present study used a quantitative survey method (Sekaran and Bougie, 2016). A cross-sectional data collecting approach was adopted using several statistical tests to assess the hypotheses. This study's purpose was to see how narcissism and sadism affect workplace incivility, with antagonism, paranoia, and emotional intelligence acting as mediators. The structured instrument was used to check the framework and hypotheses. This research used the following scales: narcissism, sadism, antagonism, and paranoia as well as EI and workplace incivility. The study's population consisted of educational institutions from Pakistan. All faculty members and teaching staff were included in the study. Non-probability convenience sampling was used for the selection of the sample. A total of 215 completed questionnaires were received and used in the analysis.

### Instruments

All items were measured on a seven-point scale, with one for “strongly disagree” and seven for “to strongly agree”. The following measuring instruments were used for data collection.

#### Narcissism and sadism

Narcissism and Sadism both were measured on 27 items using measuring instrument Short Dark Tetrad (SD4) (Paulhus et al., 2021) consisting of seven items for narcissism and seven for sadism, respectively.

#### Instigated workplace incivility

Workplace incivility was measured through Instigated Workplace Incivility scale adopted from Jiménez et al. (2018), consisting of eight measuring items.

#### Antagonism

We measured antagonism by using a super-short form of the Five-Factor Narcissism Inventory (FFNI-SSF) contains eight measuring items adopted from West et al. (2021).

#### Paranoia

Data regarding paranoid personality disorder (PPD) was gathered using a brief measure of paranoid thoughts developed by Bianchi and Verkuilen (2021) having eight measuring items.

#### Emotional intelligence

We measured EI through 10 measuring items developed by Davies et al. (2010).

### Data analysis tools and techniques

Analysis of data was carried out using PLS-SEM (partial least square structural equation modeling) (Ringle et al., 2015). It is common for PLS-SEM to be used to analyze data that is not typical. SEM and PLS techniques were utilized to develop measurement and structural modeling (Hair et al., 2017). We employed a complicated mediation model in this study since we couldn't run a model evaluation simultaneously in SPSS while using regression. Structural equation modeling was also used for analyzing the results of the experiment. To solve problems and test models, two techniques are available: covariance-based (CB-SEM) software such as Liseral, Mplus, and AMOS-SEM, or PLS-SEM and Warp PLS. According to Anwar et al. (2020), PLS-SEM offers the following advantages: small sample sizes can be used, formative models may be studied and researched, and PLS-SEM is a better alternative for assessing advanced models like mediation models. PLS-SEM was also claimed to be the most trustworthy technique for assessing mediation models since it is not limited by sample size, normal distribution of data, or independent assumptions. Factor loadings, AVE, CR, and alpha values were used to determine the scales' validity and reliability (Hair et al., 2017).

Discriminant validity was also examined utilizing criteria (Henseler et al., 2015) and continued by other researchers. The researcher ensured that all ethical factors were taken into account (Ramayah et al., 2018). Respondents were not asked for personal information, and their identity was concealed. Employee confidentiality was ensured, and verbal participant agreement was obtained.

### Measurement and structural model

Convergence and discrimination properties of the measurement model were demonstrated by validity testing. To determine whether the items and constructs measured the same ideas, the concepts of convergence and discriminant validity were both utilized (Hair Jr et al., 2014; Ramayah et al., 2018). The hetero- and mono-trait ratios were examined to determine the discriminant validity (HTMT ratios). As stated by Black and Babin (2019), the threshold value for HTMT's cut-off points is less than one. The researcher next developed a model to test the idea (Ramayah et al., 2018).

### Demographic information of respondents

Table 1 shows demographic information of respondents. Respondents were asked about their gender and designations. A majority of the respondents were male [male 115 (53.48%), female 100 (46.51%)], with most of them in the assistant professor rank 105 (48.83%) followed by lecturers 51 (23.72%). About 46 (21.39%) respondents were associate professors and 13 (6.04%) were full professors.

**Table 1:** Demographic information of respondents.

**Variables**	***N***	**Percentage**
Male	115	53.48
Female	100	46.51
Lecturer	51	23.72
Assistant Professor	105	48.83
Associate Professor	46	21.39
Professor	13	6.04

## Results

### Measurement model

**Figure 2:**
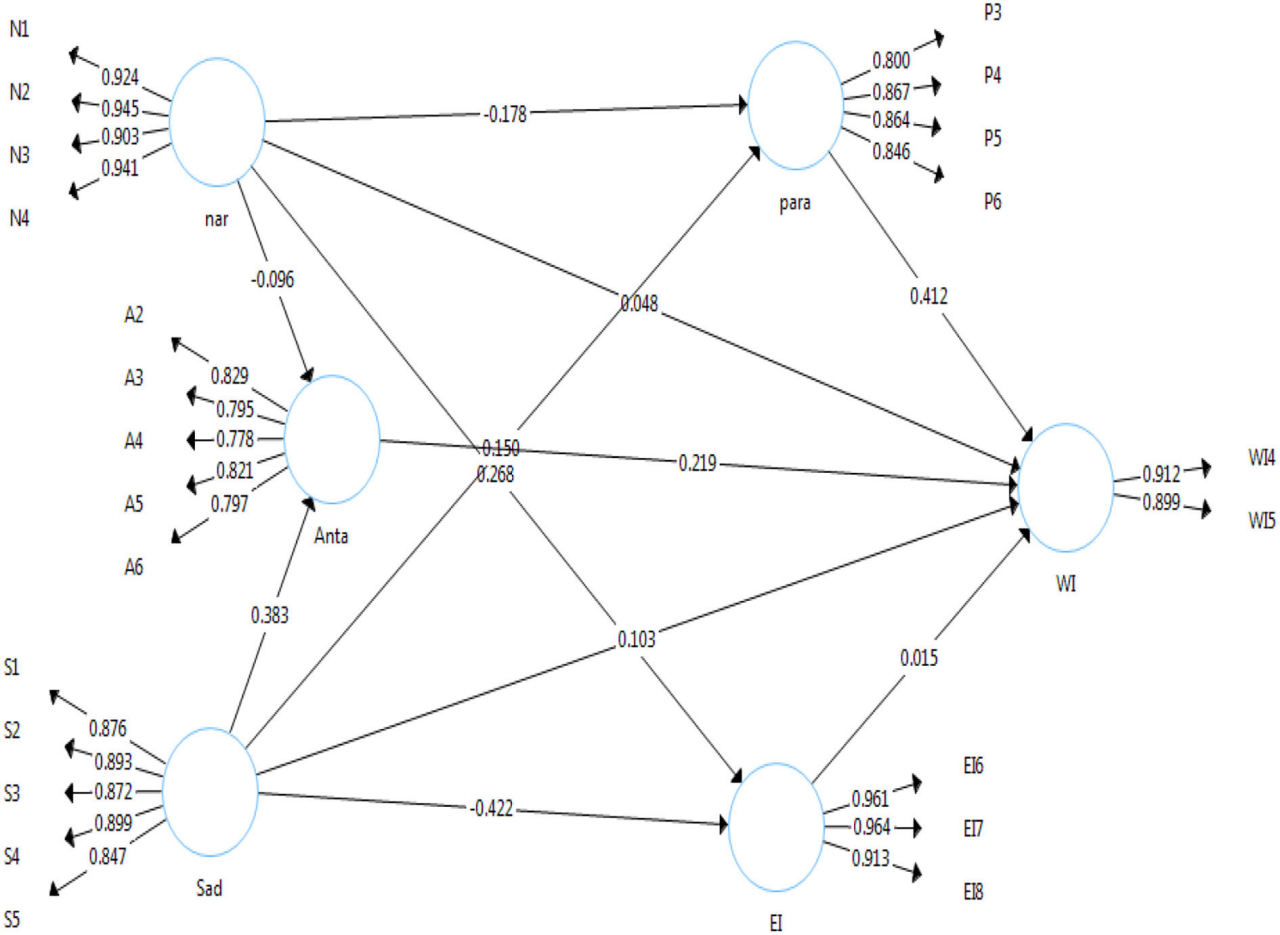
Measurement Model CFA PLS-SEM.

Guidelines to evaluate the measurement model in PLS-SEM are given by Hair Jr et al. (2020). The factor loadings should be ≥0.708, composite reliability (CR)≥ 0.700 and average variance extracted (AVE≥ 0.500) (see Figure 2 for factor loadings and Table 2 for CR and AVE). Thus, based on the values presented in Table 2, we can conclude we had sufficient convergent validity and reliability. In addition, for discriminant validity HTMT ratio we followed guidelines suggested by Franke and Sarstedt (2019). The guidelines are if the HTMT ratios are ≤0.85 then we can conclude that discriminant validity has been achieved. As shown in Table 3, all the HTMT ratios were lower than 0.85 thus the measures in our study have good discriminant validity.

**Table 2:** Descriptive and quality of measurement items.

**Constructs**	**Mean**	**Std dev**.	**Kurtosis**	**Skewness**	**CR**	**AVE**
Narcissism	3.91	1.70	−1.313	−0.045	0.961	0.861
Sadism	4.05	1.74	−1.167	−0.212	0.944	0.770
Paranoia	3.99	1.17	−1.148	−0.084	0.909	0.714
Antagonism	4.00	1.15	−0.900	−0.134	0.901	0.646
Emotional intelligence	4.01	1.36	−1.349	−0.149	0.962	0.895
Workplace incivility	3.99	1.37	−1.136	−0.041	0.901	0.820

**Table 3:** Discriminant validity.

**Constructs**	**1**	**2**	**3**	**4**	**5**
Antagonism					
Emotional intelligence	0.156				
Narcissism	0.067	0.178			
Paranoia	0.505	0.130	0.145		
Sadism	0.395	0.367	0.262	0.131	
Workplace incivility	0.527	0.112	0.034	0.612	0.259

Bootstrapping was run with a 1,000 resampling rate. The above Table 4 has presented the results of direct effects. From the findings it is evident that narcissism has an insignificant effect on workplace incivility β=0.049, *t* = 0.667, *p* > 0.05, BCILL = −0.083 and BCIUL = 0.153. Further sadism has also an insignificant effect on workplace incivility β = 0.100, *t* = 1.368, *p* > 0.05, BCILL = −0.011, and BCIUL = 0.242. Moreover, paranoia has a positive and significant effect on workplace incivility β = 0.413, *t* = 5.876, *p* < 0.05, BCILL = 0.295, BCIUL = 0.524. Further analysis of the results revealed that antagonism has a positive and significant effect on workplace incivility β = 0.221, *t* = 2.922, *p* < 0.05, BCILL = 0.090, BCIUL = 0.336. Emotional intelligence has insignificant influence on workplace incivility β = 0.013, *t* = 0.216, *p* > 0.05 BCILL = −0.097, BCIUL = 0.126, respectively. Thus, H1, H2, and H5 are not substantiated and rejected. On the other hand, H3 and H4 are substantiated and accepted.

**Table 4:** Hypotheses testing (direct effects).

**Hypothesis**	**Relationship**	**Std beta**	**Std error**	***t*** **-value**	***p*** **-value**	**BCI LL**	**BCI UL**
H1	Nar→ WPI	0.049	0.071	0.667	**0.253**	−0.083	0.153
H2	Sad→ WPI	0.100	0.076	1.368	**0.086**	−0.011	0.242
H3	Paranoia→ WPI	0.413	0.070	5.876	0.000	0.295	0.524
H4	Antag→ WPI	0.221	0.075	2.922	0.002	0.090	0.336
H5	EI→ WPI	0.013	0.067	0.216	**0.415**	−0.097	0.126

*Bold Values shows insignificant statistics*.

Indirect effects as presented in Table 5, i.e., mediating effects are investigated in PLS-SEM using 1,000 bootstrap replications. It is revealed that paranoia mediated between narcissism and sadism and workplace incivility. i.e., β = −0.075, *t* = 2.206, *p* < 0.05 BCILL = −0.135, BCIUL = −0.025, β = 0.062, *t* = 2.000, *p* < 0.05, BCILL = 0.017, BCIUL = 0.120, respectively. In addition, antagonism does not mediate between narcissism and workplace incivility β = −0.022, *t* = 1.022, *p* > 0.05 BCILL = −0.064, BCIUL = 0.004, respectively but antagonism mediated between sadism and WPI β = 0.084, *t* = 2.529, *p* < 0.05 BCILL = 0.035, BCIUL = 0.148. Emotional intelligence does not have any mediating effect between narcissism and sadism i.e., β = 0.004, *t* = 0.208, *p* > 0.05 BCILL = −0.026, BCIUL = 0.037, and β = −0.006, *t* = 0.213, *p* > 0.05 BCILL= −0.054, BCIUL = 0.044. hence H6a, H6b, H7b are substantiated while H7a, H8a, and H8b are not substantiated and rejected.

**Table 5:** Hypotheses testing (indirect effects).

**Hypothesis**	**Relationship**	**Std beta**	**Std error**	***t*** **-value**	***p*** **-value**	**BCI LL**	**BCI UL**
H6a	Nar→ Par→ WPI	−0.075	0.033	2.206	0.014	−0.135	−0.025
H6b	Sad→ Par→ WPI	0.062	0.031	2.000	0.023	0.017	0.120
H7a	Nar→ Anta→ WPI	−0.022	0.021	1.022	0.154	−0.064	0.004
H7b	Sad→ Anta→ WPI	0.084	0.033	2.529	0.006	0.035	0.148
H8a	Nar→ EI→ WPI	0.004	0.019	0.208	0.418	−0.026	0.037
H8b	Sad→ EI→ WPI	−0.006	0.029	0.213	0.416	−0.054	0.044

## Discussion

In this study, we examined how narcissism and sadism affect workplace incivility, with antagonism, paranoia, and emotional intelligence acting as mediators. The current study established the hypotheses that narcissism and sadism are positively connected with workplace incivility with the help of the contemporary integrative interpersonal theory (Sullivan, 2013) and the polyvagal theory (Porges, 1995). The current study also investigated the mediating role of antagonism, paranoia, and emotional intelligence on uncivil behavior of those who have significantly higher narcissistic and sadistic personality disorders through the lens of contemporary integrative interpersonal theory and the Polyvagal theory. This study adds to the current literature and body of knowledge about the combined influence of narcissism, sadism, antagonism, paranoia, and EI on psychological functioning as well as workplace incivility. We used a cross-sectional research design and data were obtained using previously used questionnaires. The partial least square structural equation modeling was used to investigate the hypotheses (PLS-SEM). This program can examine both measurement and structural models at the same time. In the current study, eight research hypotheses were proposed and tested.

Hypothesis 1 was established to examine the positive effect of narcissism on workplace incivility. The findings of the current study contradict H1. The findings show that narcissistic personality disorder is not to blame for workplace incivility. Self-adulation, adoration, and self-actualization make narcissism easy to deal with by professionals. These findings are in line with Morf and Rhodewalt (2001) but opposed to a few earlier studies (Meier and Semmer, 2012; Liu et al., 2020; Moon and Morais, 2022) which found that narcissistic personality characteristics such as excessive self-love, adoration, exhibition, and greater self-esteem promote counterproductive workplace behavior i.e., incivility. Meier and Semmer (2012) investigated the factors that lead to uncivil behavior toward coworkers and supervisors., job characteristics (narcissism), personality (work-related rage), and work characteristics (lack of reciprocity in the connection with one's organization) were all examined jointly. Their findings suggested that anger acted as a mediator between incivility and lack of reciprocity and that this mediation is particularly significant among narcissistic employees (moderated mediation). The study revealed that anger at least partly mediated the link between incivility and lack of reciprocity. Their findings also revealed that narcissism moderated the first half of the mediation chain (lack of reciprocity and anger), but not the second component (anger and incivility). In the study by Liu et al. (2020), narcissism had a substantial positive influence on workplace incivility, whereas anger and guilt positively mediated the association between narcissistic personality disorder and workers' incivility at work. Moon and Morais (2022) claimed that heightened narcissism can influence workplace incivility while employees' self-esteem and working norms have a key role in contributing to a destructive environment at work. In contrast, Morf and Rhodewalt (2001) observed that narcissistic traits were not correlated with incivility toward coworkers but were associated with incivility against supervisors in a marginally significant manner. So, based on the above discussion H1 is rejected.

Hypothesis 2 was established to examine the positive effect of sadism on workplace incivility. The findings of the current study did not support this hypothesis by establishing the insignificant effect of sadism on workplace uncivil behavior and explaining that social detachment and pleasure in cruelty did not predict incivility at work. These findings are opposed to those of others (Thibault and Kelloway, 2016; Min et al., 2019), whereas Thibault and Kelloway (2016) found that sadism has a muted influence on the dark triad and counterproductive workplace behavior and that the dark triad lost its predictive power over CWB when the sadism score is low. These findings also revealed that sadism might play a role in the establishment of other negative personality characteristics at work, with a greater link to workplace expression. Similarly, Min et al. (2019) found that sadism (a dispositional motivation that drives offenders to participate in workplace maltreatment) increases the prevalence of cyberbullying over the other two (interpersonal deviance and inspired incivility). Both studies concluded that sadism is the root of all uncivil behavior at work. Thus, based on the above discussion H2 is also rejected.

Hypothesis 3 was established to investigate the impact of paranoia on workplace incivility. We hypothesized that paranoid personality disorder (PPD) would have a significantly positive impact on workplace incivility. The current research findings confirm the study's premise. Clarifying that deliberate paranoid thinking with heightened distrust, fear of being assaulted or harassed at work (i.e., paranoid arousal), and the perception of being harmed or harassed at work are some particular causes of workplace incivility. These findings are consistent with those of Lopes et al. (2020) and Finn and Constable (2022) who explained that paranoid thinking was not only common among the UK and French teachers but was also associated with bullying perceptions and intentions to participate in workplace misconduct. Furthermore, it was found in the same study that neither negative mood nor workplace bullying mediated the relationship between supervisory paranoia and a willingness to engage in workplace misbehavior, but that the relationship between supervisory paranoia and a willingness to engage in workplace misbehavior was only mitigated due to past stressors and harmful psychological experiences i.e., paranoid beliefs. Although Finn and Constable (2022) found that social functioning can be negatively impacted by varying levels of paranoid severity. Further, when it comes to stigmatized employment conditions, Mitelman et al. (2020) observed that this may be especially true for workers with paranoia. They also indicated that one's ability to be attentive is essential for the insidious process linking social stress to paranoid cognition and, as a result, lower job wellbeing. In the current study, similar findings were seen. H3 is accepted based on the preceding debate.

Hypothesis 4 was established to examine the positive effect of antagonism on workplace incivility. Our hypothesis predicted that antagonism personality disorder had a significantly positive impact on workplace incivility. The current research findings confirm the study's premise. Clarifying that individuals with low agreeability or strong antagonistic qualities are shown to have a distrustful, dishonest, greedy, uncooperative, uncivil, and haughty attitude toward their workplace environment, the findings of the current study are in line with previous studies (Hurtz and Donovan, 2000; Shiverdecker and LeBreton, 2019; Hall et al., 2021). Hurtz and Donovan (2000) found a negative association between antagonism and job performance, indicating that antagonistic personality features were a stronger precursor of promoting workplace negligence. Shiverdecker and LeBreton (2019) argued that antagonistic personality disorder can promote workplace uncooperative behavior and uncivil socialization. On the other hand, Hall et al. (2021) conducted research by associating the two key subcomponents of externalizing—antagonism and disinhibition—with particular control processes through the use of a battery of inhibitory control tasks and accompanying computer modeling. They revealed that antagonism was related to particular deficiencies in quick inhibitory control processes involved in withholding prepared/prepotent responses and filtering distracting information. Disinhibition and temporary anxiety, on the other hand, were linked to workplace aggression rather than job performance. Hence H4 is also acceptable based on the preceding debate.

Hypothesis 5 was established to examine the negative effect of EI on workplace incivility. Our hypothesis posits that EI has a negative effect on workplace incivility. Results were found to have insignificant associations. Meaning that individuals who have a high level of emotional intelligence can better control their emotions and prevent impulsive actions that might hurt their coworkers. Results from this study were consistent with Ramsey-Haynes (2021) who examined oncology nurses' EI and workplace incivility. Workers with strong emotional intelligence were shown to be insignificantly associated with workplace incivility and workplace misbehaviors. The workplace culture benefits from high EI and low incivility, yet people usually lack self-awareness about their behaviors. The study also revealed that the nurses with improved emotional intelligence (EI) were better able to connect with their patients and coworkers on a deeper level. Thus, H5 is rejected.

Hypotheses 6a and 6b were developed to examine whether paranoia mediated between narcissism and workplace incivility, and sadism and workplace incivility. These hypotheses were found to be significant in the study findings. From the results, it was observed that paranoia positively and significantly mediated the relationship between narcissism and workplace incivility, explaining that narcissists regard uncivil conduct as a danger to their objective of a positive self-image and are prompted to protect themselves because of their high self-enhancement desire. As a result, narcissists are more likely to feel rage and less remorse, because they can preserve a favorable self-image by blaming incivility on other reasons rather than their own defects. If persons in the previous or current working environment have encountered the same psychological impairment pattern of paranoid triggers, this impact might become more pronounced. These results are consistent with Sofra (2020) and Bani-Melhem et al. (2020). Both studies revealed that narcissists with paranoid tendencies are hypervigilant and suspicious at work, which helps them identify and anticipate hidden rivals or hazards in their surroundings. A dark personality and paranoia, in addition to personal resources, may prohibit people from performing their duties properly. Furthermore, given the mediating impact of paranoia, identical findings were obtained in cases of sadism and workplace incivility. That is, sadistic impulses in combination with paranoid features might foster uncivil behavior in the workplace. Sadism looks to be very flexible incivility with increased paranoid trauma due to its neurotic character. The absence of depressive symptomatology in their profiles, on the other hand, suggests that elite paranoid personalities may respond to adversity with sadism. These results were in line with (Sofra, 2020). Hence study hypotheses H6a and H6b are accepted and substantiated.

Hypotheses 7a and 7b were established to examine whether antagonism mediated narcissism, sadism, and workplace incivility. From the results, it was found that antagonism has insignificantly mediated between narcissism and workplace incivility, indicating that due to their high degrees of self-enhancement desire, strong self-esteem, and self-centeredness, antagonistic narcissist personalities exhibited decent behavior in their workplace socializing. These results are opposed to the findings of Rózycka-Tran et al. (2015) and Lynam and Miller (2019). In the study by Rózycka-Tran et al. (2015), it was shown that open rivalry with others was linked to antagonistic narcissism. Clarifying that antagonism should be linked to societal convictions that have been tied to a negative perspective of interpersonal connections, which indicates conflict in people's interests. This way of thinking is adversely correlated with trust, which eventually encourages workplace incivility. Similarly, Lynam and Miller (2019) claim that antagonistic propensities are mostly caused by externalizing behavioral features such as antisocial personality disorder (APD) and narcissistic personality disorder (NPD). Both studies revealed that narcissists with antagonistic tendencies are hypervigilant and suspicious at work, which makes them have strong opposition in a workplace environment. Even more so, antagonism and sadism appear to have a mediated influence on workplace incivility because of the antagonism-induced mediation of sadistic impulses and antagonism traits. To put it another way, sadists who have high levels of antagonism disorder and low levels of self-control, and an addiction trauma for intrinsic pleasure at the cost of others' misfortune can lead to constant workplace incivility. High antagonistic persons may respond to incivility because of their lack of empathy and desire for pleasure. These results are in line with previous research (Tiedens, 2001; Foulkes, 2019; Beckert and Ziegele, 2020). Research by Beckert and Ziegele (2020) found that those with sadistic personality traits were more likely to exhibit an incivility-inducing attitude, whereas those with more pleasant personality traits were more likely to exhibit an attitude of deliberation. Sadism, whether sexual or non-sexual, may occur with antagonistic personalities, and Foulkes (2019) stated that sadism with hedonistic enjoyment should be examined with antagonistic personality qualities, rather than antisocial, poor self-control, and impolite feature. Conversely, Tiedens (2001) discovered that grief and dread are highly connected with workplace incivility and that this conduct might have a detrimental impact on individuals and organizations. The effects of fear and grief are enhanced when individuals have a lower status. These findings emphasized the need for enhancing public understanding of incivility and its effects, as negative consequences may be obscured depending on the individual's status and contextual variables. Ultimately overt competition with others is associated with antagonistic sadism. Thus, H7a is rejected while H7b is accepted.

Hypotheses 8a and 8b were established to examine whether emotional intelligence had negatively mediated narcissism, workplace incivility, sadism, and workplace incivility. From the results, it was found that emotional intelligence has insignificantly mediated the relationship between narcissism and workplace incivility. Explaining that EI has no role in workplace incivility of narcissistic personalities, these results were in line with Jonason and Krause (2013) while opposing the study findings of Petrides et al. (2011), Veselka et al. (2012), and Karim et al. (2015) which observed a limited but significant association between narcissism and EI. Jonason and Krause (2013) observed that those with narcissistic personalities showed low affective empathy and had trouble picking up on the feelings of others i.e., emotional intelligence. Social and emotional skills are not always used by individuals having higher narcissism and counterproductive working behavior to deceive others. Finally, with the mediating effect of EI, the same results were observed in cases of sadism and workplace incivility. Sadism and workplace incivility were found to have a negligible effect on EI. Explaining that sadistic feelings and behaviors like dominance, ridicule, enslaving, biting, scorching, flogging, invasion, suffocation, and physical mutilation are not regulated by emotional intelligence. The present study's findings are opposed to those of the previous study by Rastogi and Shukla (2021), where they observed that non-delinquents were emotionally more sophisticated than delinquents, and delinquents had more sadistic inclinations than non-delinquents. As a result, H8a and H8b are rejected.

## Conclusions

Negative behaviors at work are common but do great harm to the organizations. Employees and managers are badly affected by negative attitudes such as sadism, and paranoia antagonism, which lead to workplace incivility. Individuals having a narcissistic personality love their work and thus do not create any negative situations at work. On the contrary, individuals having attributes of sadism, paranoia, and antagonism create negative situations which lead to incivility. Given that emotional intelligence could play an important role to reduce negative behaviors, managers must take advantage of it and help reduce negative behaviors such as sadism, paranoia, and antagonism. Managers must encourage teamwork and supportive culture in the workplace so that team members support each other to achieve organizational targets on time as well as personal growth and career development.

### Theoretical and managerial implications

This is an original work that has contributed to a body of knowledge by extending the literature on narcissism, sadism, paranoia, antagonism, emotional intelligence, and workplace incivility through the lens of contemporary integrative interpersonal theory and the Polyvagal theory. This scientific work empirically tested the framework given in the manuscript by successfully adding three parallel mediators by determining parallel mediating effects on the relationship between narcissism, sadism, and workplace incivility. Secondly, the existing study has implications for managers and policymakers. Managers should discourage those negative behaviors which are harmful to the workplace and the image of the firm. They should encourage teamwork, supportive culture, and make formal teams, committees, and groups which help people to work together and learn to work in a team to achieve organizational objectives and obtain an advantage of working in a group.

### Limitations and future recommendations and directions for research

The current study has offered several contributions which are discussed above but some limitations are essential to address here so future studies may cover them. The very first limitation is the single method of data collection and analysis which may lead to common method bias (CMB) and common method variance (CMV). According to Creswell and Zhang (2009), the single method might lead to biasness so it is recommended to use mixed methods such as quantitative and qualitative data so more in-depth and a better understanding of the subject matter may be obtained. On the other hand, longitudinal data could also be used. The second limitation is the data was collected from one sector, so one must be careful while generalizing the findings to another sector. Third, three mediators are used in the current study. In the future, the same model can be applied by adding other mediators and moderators such as supportive culture, team spirit, and servant leadership style to reduce workplace incivility.

## Data availability statement

The raw data supporting the conclusions of this article will be made available by the authors, without undue reservation.

## Ethics statement

Ethical review and approval was not required for the study on human participants in accordance with the local legislation and institutional requirements. Written informed consent from the patients/participants or patients/participants' legal guardian/next of kin was not required to participate in this study in accordance with the national legislation and the institutional requirements.

## Author contributions

BW contributed to the data curation. MF, IU, and BW contributed to the revision of the manuscript. YM contributed to the supervision and guidelines. AK contributed to the formatting of the manuscript. IU contributed to the conceptualization. IU and BW contributed to formal analysis and original draft. AK and WW contributed to the review. WW contributed to the writing and editing of the manuscript. All authors contributed to the article and approved the submitted version.

## Funding

This research was fully funded by Beijing Philosophy and Social Science Foundation Project (21GLC057).

## Conflict of interest

The authors declare that the research was conducted in the absence of any commercial or financial relationships that could be construed as a potential conflict of interest.

## Publisher's note

All claims expressed in this article are solely those of the authors and do not necessarily represent those of their affiliated organizations, or those of the publisher, the editors and the reviewers. Any product that may be evaluated in this article, or claim that may be made by its manufacturer, is not guaranteed or endorsed by the publisher.
